# New-Onset Diabetes with Ketoacidosis Precipitated by COVID-19 in Children: A Report of Two Cases

**DOI:** 10.1155/2021/5545258

**Published:** 2021-07-16

**Authors:** Nabila Chekhlabi, Amal Haoudar, Nadia Echcharii, Said Ettair, Nezha Dini

**Affiliations:** ^1^Department of Paediatrics, International University Hospital Cheikh Khalifa, Mohammed VI University of Health Sciences UM6SS, Casablanca, Morocco; ^2^Department of Intensive Care Unit, International University Hospital Cheikh Khalifa, Mohammed VI University of Health Sciences UM6SS, Casablanca, Morocco; ^3^Mohamed V University, Faculty of Medicine and Pharmacy of Rabat, Rabat, Morocco

## Abstract

**Background and Aims:**

There is growing evidence that the 2019 coronavirus disease (COVID-19) is emerging as a potential trigger virus for the development of diabetes mellitus in children. This can occur even in patients without factors predisposing to impaired glucose metabolism. Here, we report two rare cases of diabetic ketoacidosis revealing new-onset diabetes and precipitated by COVID-19. These cases are reported in view of their rarity and originality. The relationship between type 1 diabetes mellitus and COVID-19 is discussed.

**Results:**

Two children developed symptoms suggestive of diabetic ketoacidosis preceded by polyuria, polydipsia, and asbestos. There is a documented COVID-19 infection in the parents of the 2 children. An asymptomatic infection was detected in the 2 patients on the basis of a reverse transcription polymerase chain reaction (RT-PCR) test. Thoracic imaging and inflammatory workup were negative in both cases. Both patients responded well to treatment, including rehydration regimens and intravenous insulin. On the 2nd day of their hospitalization, they were transferred to several injections of subcutaneous insulin with therapeutic and nutritional education from the parents. After about 4 weeks, their insulin requirements probably decreased due to the diabetes honeymoon.

**Conclusion:**

COVID-19 can induce acute onset diabetes and diabetic ketoacidosis in children. More research data are needed to improve our knowledge of this constellation and to guide the most appropriate therapies.

## 1. Introduction

The pandemic secondary to the 2019 novel coronavirus disease (COVID-19) has resulted in an unexpected loss of life and a change in quality of life and economy. The clinical spectrum of COVID-19 varies from mild forms to death. COVID-19 has unpredictable effects on many organs. The impact of COVID-19 on endocrine systems is understudied, and its specific metabolic complications are not yet fully understood. Since the start of this pandemic, a large number of published scientific reports have almost all shown that patients with diabetes mellitus (DM) face a more severe form of COVID-19 with a high death rate [1]. All over the world, a high prevalence of diabetic ketoacidosis revealing type 1 diabetes on COVID-19 infection has been reported in children and also in adults. Interestingly, “new-onset” hyperglycemia and “new-onset” DM are now emerging as probable complications of COVID-19, especially among hospitalised patients. This suggests the hypothesis that SARS-CoV-2 infection could either precipitate a new type of DM by destruction of pancreatic cells or could trigger new pathophysiological mechanisms linked to MD [2]. Such a hypothesis requires large multicenter studies to confirm it.

Here, we report the case of two children with acute diabetes and diabetic ketoacidosis, precipitated by coronavirus disease 2019 (COVID-19); they were treated and then followed for 4 weeks to assess the balance of diabetes. The detailed history, anthropometry, laboratory tests, imaging studies, treatment administered, and clinical course have been documented.

## 2. Case Report

### 2.1. Case 1

A 4-year-old girl, with no specific pathological history, was admitted to the hospital with somnolence and polypnea. The symptoms started 15 days before with a slight flu-like syndrome and then by polyuria, polydipsia, and polyphagia contrasted with a weight loss of 3 kg and complicated, 2 days before admission, by fatigue, abdominal pain, vomiting, and polypnea. There is no family history of type 1 diabetes (T1D) nor autoimmune conditions. After further investigation, both parents admitted to having a positive COVID-19 diagnosis a week ago. At admission, she was haemodynamically stable but mildly tachycardic and drowsy without neurological deficit. She presented with severe dehydration and had an ample tachypnea of acidosis (Kussmaul's breathing) with normal saturation at 98%. Her temperature was 37.5°C, weight was 16.5 kg, and length was 106 cm. The remainder of the clinical examination did not find any source of infection. Her capillary blood sugar was 5.8, and the urine strips showed 3 sugar crosses and 3 acetone crosses. This is in favour of inaugural diabetic ketoacidosis. The initial laboratory assessment, summarized in Table 1, noted hyperglycemia, acidosis, normal blood count, negative CRP, and negative PCT. The ionogram noted hyponatremia at 128 mEq/L and a low alkaline reserve at 2 mmol/L. HbA1c was elevated to 11.8%. Islet cell antibodies and antibody glutamic acid decarboxylase were positive. Chest X-ray was normal except for bronchial syndrome. Nasopharyngeal COVID-19 PCR and SARS-CoV-2 antibody test returned positive.

She received rehydration and intravenous insulin therapy with correction of hyponatremia and close clinical monitoring. Correction of DKA was achieved in approximately 24 hours after the start of treatment. On the 2nd day, the diet was started with 3 main meals and a snack, and intravenous insulin was replaced by subcutaneous insulin (Lantus (glargine) in the form of basal insulin and Novorapid (insulin aspart) as mealtime insulin). Blood glucose was regularly monitored, and parent diabetes education was provided. No specific COVID-19 treatment was given, and the control COVID-19 PCR came back negative on day 7 of hospitalization. Eight days after her hospitalization, she was discharged with a diagnosis of new-onset type 1 diabetes and a concomitant COVID-19 infection.

### 2.2. Case 2

A 7 years and 10 months previously asymptomatic boy presented to the emergency department with inaugural diabetic ketoacidosis. Symptoms started 3 weeks ago with fatigue, polydipsia, polyuria, and weight loss of 3 kg and complicated by vomiting, abdominal pain, and severe polypnea. On questioning, we found the notion of a recently confirmed COVID-19 infection in the father. On admission, the patient was conscious, very asthenic, and dehydrated but haemodynamically stable. He also presented with moderate tachypnea, indicative of metabolic acidosis. He weighed 22 kg with a correct height at 121 cm. His capillary blood sugar was 4.5, and urine analysis showed a large amount of sugar and ketone. The initial laboratory assessment, summarized in Table 1, noted hyperglycemia, acidosis, normal blood count, negative CRP, and negative PCT. The ionogram noted hyponatremia at 130 mEq/L and a low alkaline reserve at 6.8 mmol/L. HbA1c was elevated to 10.3%. Insulin antibodies, islet cell antibodies, and antibody glutamic acid decarboxylase were negative. Screening SARS-CoV-2 RT-PCR returned positive. The chest X-ray was normal. He received intravenous rehydration followed by intravenous insulin therapy with correction of hyponatremia and close blood glucose monitoring. The ACD corrected in about 18 hours after starting treatment. After stabilization, intravenous insulin therapy was replaced by subcutaneous insulin (Lantus (glargine) as basal insulin and Novorapid (insulin aspart) as premeal insulin), an appropriate diet was started, and therapeutic parent education was provided. Given the apyrexia and the absence of a respiratory sign, no specific COVID-19 treatment was administered, and the COVID-19 PCR control returned negative on day 8 of hospitalization. He was released after ten days of hospitalization. The patient was seen again for control after a week and then after a month. He presented with hypoglycaemia following which the insulin doses were reduced with good progress.

## 3. Discussion

Globally, from its outbreak in December 2019 through January 1, 2021, 81,658,440 confirmed cases of COVID-19, including 1,802,206 deaths, have been reported to the WHO [3]. An epidemiological study conducted in China showed that 90% of 731 laboratory-confirmed COVID-19 patients under the age of 18 had asymptomatic, mild, or moderate infection, making it relatively mild than adult infection [4]. Type 1 diabetes mellitus (T1DM) or type 2 diabetes mellitus (T2DM) and underlying cardiovascular disease are considered risk factors for increased disease severity and mortality from coronavirus 2019 (COVID-19) [5].

Diabetic ketoacidosis (DKA) is a metabolic disorder caused by total or partial insulin deficiency that can be life-threatening in the absence or delay in treatment [6]. It is more common in children under 5 years old. DKA is defined biochemically as venous pH < 7.3 or serum bicarbonate concentration <15 mmol/L, serum glucose concentration >11 mmol/L (>200 mg/dL) associated with ketonemia, glycosuria, and ketonuria [7]. Information regarding the inaugural occurrence of type 1 diabetes during COVID-19 infection in children is evolving rapidly as data continue to emerge around the world. In a retrospective study in China, 42 (6.4%) patients admitted with COVID-19 had ketosis, of which only 15 (35.7%) had known diabetes [8]. A Germany study found a significant increase in diabetic ketoacidosis and severe ketoacidosis at diabetes diagnosis in children and adolescents during the COVID-19 pandemic [9]. The causes of this increase can be multifactorial and can be explained by reduced medical services and the fear of COVID-19 contamination and therefore of approaching the healthcare system [9].

Infections are often considered the most common triggering factor for DKA in known or inaugural diabetes. It is believed that certain viral diseases can precipitate type 1 autoimmune diabetes in genetically predisposed patients [10]. New-onset hyperglycemia has been described as the result of various infections, including HIV [11]. Serologic evidence of infection and viral isolation in the pancreas has been reported in a few cases of newly diagnosed diabetes [10, 12]. Interactions between COVID-19 infection and the renin-angiotensin-aldosterone system (RAAS) could explain the pathophysiology of DKA. The presence of angiotensin-converting enzyme 2 (ACE2) in significant amount in the endocrine part of the pancreas suggests that the coronavirus enters the islets using ACE2 as a receptor and causes destruction of these islets leading to acute diabetes mellitus [13]. In addition, the aberrant immunity elicited by SARS-CoV-2 may induce autoimmune destruction of pancreatic islet cells mimicking the pathogenesis of insulin-dependent diabetes [14].

The impact of SARS-CoV-2 on the RAAS also has clinical implications for the treatment of DKA. Fluid infusion should be done with caution to avoid worsening the acute respiratory distress syndrome as angiotensin increases pulmonary vascular permeability and potentiates lung parenchyma damage [15].

Interestingly, there is a case series of two toddlers who developed insulin-dependent diabetes with DKA a few months after the diagnosis and treatment of Kawasaki disease [16]. These cases support the relationship between postinfection COVID-19 inflammation and pancreatic endocrine dysfunction leading to diabetes.

However, no one can confirm whether blood sugar disorders secondary to COVID-19 disease persist or go away when the infection clears. Does COVID-19 unmask the silently existing DM rather than induce the new DM in these patients? To answer these questions and better understand the mechanisms described, an international group of diabetes researchers participating in the CoviDIAB project established a global registry of patients with diabetes linked to COVID-19 in order to study the phenotype of new-onset diabetes linked to a COVID-19 infection [2].

Finally, even if children do not have the classic presentation of SARS-CoV-2 pneumonia like adults, a high degree of vigilance is needed, in any child who is positive or living in a family cluster, to early detect new diabetes before diabetic ketoacidosis and therefore improve the prognosis of metabolic complications related to COVID-19.

## 4. Conclusion

These observations support the hypothesis of a potential diabetogenic effect of COVID-19 in addition to the stress response often associated with serious illness. COVID-19 can also unmask existing DM by worsening its metabolic complications in some patients. Extensive additional research is needed to confirm the reality of this relationship.

## Figures and Tables

**Table 1:** The main biological assessments of the 2 patients.

Measure	Reference range	Case 1	Case 2
White cell count (per *μ*L)	4000–145,000	6070	12,300
Neutrophil count (per *μ*L)	1500–8000	3960	9239
Lymphocyte count (per *μ*L)	1000–7000	1580	2480
Platelet count (per *μ*L)	150,000–450,000	188,000	378,000
Hematocrit (%)	M: 42–50, F: 37–47	29.6	27.8
HbA1c (%)	<6.5	11.8	10.3
Sodium (mEq/L)	136–145	128	130
Potassium (mEq/L)	3.4–4.5	3	2.7
Chloride (mEq/L)	98–107	105	115
Blood urea (g/L)	0.10–0.50	0.23	0.15
Creatinine (mg/dL)	3.2–6	2.8	2.6
Alkaline reserve (mmol/L)	25–28	2	6.8
D-dimer (*μ*gFEU/mL)	Under 0.50	0.49	0.36
Ferritinemia	15–80	92	255
CRP (mg/l)	0.1–2.8	1.9	0.1
Plasma glucose (mg/dL)	72–125	390	423
Islet cell antibodies (anti-IA2) UI/ml	Under 28	Positive (280)	Negative (8)
Antibody glutamic acid decarboxylase (anti-GAD)	Under 17	Positive (59)	Negative (3)
SARS-CoV-2 antibody test	Qualitative result	Positive	Positive
Nasopharyngeal COVID-19 PCR	Qualitative result	Positive	Positive
